# The Relationship of TPOAb and TGAb with Risk of Thyroid Nodules: A Large Epidemiological Study

**DOI:** 10.3390/ijerph14070723

**Published:** 2017-07-05

**Authors:** Weimin Xu, Liangliang Huo, Zexin Chen, Yangmei Huang, Xingyi Jin, Jing Deng, Sujuan Zhu, Yunxian Yu

**Affiliations:** 1 Department of Endemic Diseases Control and Prevention, Hangzhou Center for Disease Control and Prevention, Hangzhou 330021, China; xwmdm@sohu.com (W.X.); huoliang217@163.com (L.H.); huang-yangmei@163.com (Y.H.); jxy1499256186@163.com (X.J.); jhhydengjing@163.com (J.D.); zsj064@163.com (S.Z.); 2 Department of Epidemiology & Health Statistics, School of Public Health, Zhejiang University, Hangzhou 310002, China; chenzexin@zju.edu.cn

**Keywords:** thyroid autoantibody, thyroid nodule

## Abstract

*Objective:* The association between thyroid hormones, thyroid autoantibodies, and thyroid nodules are still not clear. The cross-sectional study, conducted in Hangzhou, China in 2010, aimed to identify the relationship of thyroid hormones and autoantibodies with thyroid nodules. *Methods*: Information regarding social demography was collected by a questionnaire. Thyroid hormones (triiodothyronine, thyroxin, free triiodothyronine, free thyroxin, thyrotropin), thyroid autoantibodies (thyroid peroxidase antibody, antithyroglobulin antibody), and thyroid nodules (diagnosed by ultrasonography) was measured in 1271 adults. The association of thyroid hormones and thyroid autoantibodies with thyroid nodules was evaluated using multiple logistic regression models. *Results:* The prevalence of thyroid nodules among males and females was 29.49% and 33.15%, respectively. The thyroid hormone level in the thyroid nodules group was significantly higher than the non-nodules group (all *p* values < 0.05), except reversely in TSH (thyroid stimulating hormone) (*p* = 0.0532) and TGAb (thyroglobulin antibody) (*p* = 0.0004). High levels of TPOAb (thyroid peroxidase antibody) (OR (Odds Ratio) = 1.51, 95% CI (confidence interval): 0.99–2.30) and TGAb (OR = 2.86, 95% CI: 1.49–5.51) were associated with increased risk of thyroid nodules, compared with corresponding low levels. However, following sub-analyses in two genders, the similar associations were only observed in females (TPOAb: OR = 1.63, 95% CI: 0.99–2.68; TGAb: OR = 3.13, 95% CI: 1.53–6.40). *Conclusions*: The present study indicated that thyroid autoantibodies were positively associated with the risk of thyroid nodules in Chinese coastal adults.

## 1. Introduction

Thyroid nodule, as an entity, is one of the most common diseases originating from the endocrine system. Thyroid nodules may be single, multiple, solid, or cystic and may or may not be functional. The thyroid epithelial cells, induced by random mutations or rearrangements, will grow from a normal state to an abnormal state. This induction of growth exacerbates cellular mutagenesis that generates the nodules [1,2]. Most thyroid nodules are benign tumors and 5% are reported as malignant [3,4].

In humans, there is an age-dependent increase in thyroid nodularity. The degree of this increase depends upon several factors, including iodine intake and its detection methods [5]. Although no evidence has proved that a benign thyroid nodule diagnosed appropriately will progress to be malignant, and the usual options include follow-ups to detect nodules changes and thyroid function, yet precise diagnosis is quite important as the incidence rate of thyroid nodules is progressively increasing. Serum thyroid hormones and thyroid ultrasonography are pivotal in evaluating thyroid nodules.

Traditionally, clinical factors such as age, gender, and radiation history are meaningful for predicting thyroid nodules. Few studies explore the association of serum indexes of thyroid hormones or autoantibodies with the risk of thyroid nodules. Recently, Haymart M.R. et al. [6] and Boelaert K. et al. [7] presented serum thyrotropin (TSH) concentration as a possible reliable predictor of malignant thyroid nodules. However, Hurtado-López L.M. et al. [8] reported that only 30.43% of the patients studied had subnormal TSH. Although thyroid hormones and autoantibodies are reported to be dependently associated with thyroid function and thyroid diseases, little attention has been paid to whether thyroid hormones and autoantibodies are associated with thyroid nodules. Meanwhile, the transformation trends between thyroid hormones or autoantibodies and thyroid nodules remain poorly understood. The underlying connection could help predict thyroid nodules in order to make more precise diagnoses. Therefore, this study was undertaken to determine whether there is an association between thyroid nodules and thyroid hormones, or thyroid autoantibodies, and to explore the transformation trends among the Chinese coastal population.

## 2. Materials and Methods

### 2.1. Population Features

This study was conducted in Hangzhou, Zhejiang Province, China, which is located in eastern China. This city includes eight districts and five counties. There are 8.7 million residents in Hangzhou city; 73.25% of them living in urban areas, 26.75% in rural areas.

### 2.2. Subjects and Study Design

A stratified, with probability proportionate to size procedure, multistage cluster sample was used to obtain a representative sample of the population. Subjects and samples were part of our previously reported study [9]. The data collection, sample measurement, and variable definitions were described in the reported study. First, three sub-districts were selected randomly from Xiacheng district. Second, one community was randomly selected from each sub-district. Third, 100 households from each community were randomly selected. Finally, 300 households were recruited for the interview. In the epidemiological study conducted from 2009 to 2012, we included 1271 residents living in Hangzhou region more than 5 years; 529 males and 742 females. The inclusion criterion was being 6 years of age or older. Subjects with any of the following characteristics were excluded from the study: (1) undergoing radioactive iodine therapy; (2) evaluated with radiologic tests using contrast media in the past 6 months; (3) taking the medicine of amiodarone; (4) having renal or other serious diseases. Only participants aged ≥18 years were included in the present manuscript.

The eligible family members of selected households were convened at a community administration center. The researchers introduced the study protocol and obtained written informed consent form from each participant. Meanwhile, an interview schedule was appointed to participants. The study protocol was approved by the Institutional Review Board of Hangzhou Center of Disease Control and Prevention (2010-001). This survey was carried out by well-trained personnel (including community clinic physicians, nurses, and public health doctors).

### 2.3. Thyroid Morphology and Ultrasonography

Anthropometric and physical examination data were obtained from each participant. Weight and standing height were measured by standardized methods, respectively. Body mass index (BMI) was calculated as weight (kg)/height (m^2^). An ultrasound examination of the thyroid was performed to detect thyroid nodules, using a Sonoline Versa Pro (Siemens, Munich, Germany) with a 7.5 MHz, 70 mm linear transducer (effective length, 62 mm). The criteria for a normal thyroid ultrasound were a homogeneous echogenic pattern throughout the gland, absence of nodules and cysts, and an absence of diffuse or heterogeneous abnormalities. Additional ultrasound structural focal abnormalities were described as nodules (circumscribed areas of greatly reduced or absent echogenicity).

### 2.4. Hormonal Measurements and Thyroid Autoantibodies

Thyroid function tests were measured by electrochemiluminescence immunoassay (ECLIA) ADVIA Centaur^®^ XP (SIEMENS, Erlangen, Germany). Thyroid function tests: Serum T3 normal values 0.60–1.81 μg/L; T4 normal values 45.0–109.0 μg/L; FT3 normal values 2.30–4.20 ng/L; FT4 normal values 8.9–17.6 ng/L; TSH normal values 0.35–5.50 mU/L; TPOAb normal values 0.0–60.0 KU/L; TGAb normal values 0.0–60.0 KU/L.

### 2.5. Unrestricted Cubic Splines

Cubic splines are generally defined as piecewise-polynomial line segments whose function values and first and second derivatives agree at the boundaries where they join. The boundaries of these segments are called knots, and the fitted curve is continuous and smooth at the knot boundaries [10]. In 2011, Nicola Orsini [11] introduced the new postestimation command “xblc” (STATA software, College Station, TX, USA), and they focused on cubic-spline logistic regression for predicting the occurrence of a binary response. Using Akaike’s information criteria (a summary measure that combines fit and complexity), they found that the unrestricted and right-restricted cubic-spline models have a better fit (smaller Akaike’s information criteria) compared with the left- and both-tail restricted cubic-spline models. Hence, in the present manuscript, we reference the method of unrestricted cubic splines in STATA.

We also show (see Supplemental Figures S1a, S2a, S3a, S4a, S5a, S6a, S7a) unrestricted cubic splines with unadjusted odds ratios plotted for each level of thyroid hormones and autoantibodies. We used an odds ratio of 1.0 to determine the cutoff value for dichotomization. The regression analysis revealed that the risk of T3 began to change at 1.3 μg/L, FT3 at 3.4 ng/L, T4 at 100 μg/L, FT4 at 14 ng/L and 20 ng/L, TSH at 3.5 mU/L and 5.5 mU/L, TPOAb at 90 KU/L, and TGAb at 400 KU/L. Therefore, the different levels of definition of thyroid hormones and autoantibodies are represented by the following: low T3: ≤1.3 μg/L, high T3: >1.3 μg/L; low FT3: ≤3.4 ng/L, low FT3: >3.4 ng/L; low T4: ≤100 μg/L, high T4: >100 μg/L; low FT4: ≤14 ng/L, medium FT4: >14 ng/L and <20 ng/L; high FT4 ≥20 ng/L; low TSH: ≤3.5 mIU/L; medium TSH: >3.5 mIU/L and <5.5 mIU/L; high TSH: ≥5.5 mIU/L; low TPOAb: ≤90 KU/L; high TPOAb: >90 KU/L; low TGAb: ≤400 KU/L, high TGAb: >400 KU/L. Graphs (see Supplemental Figures S1b, S2b, S3b, S4b, S5b, S6b, S7b) show unadjusted ORs with 95% CI (capped spikes) for the relation of thyroid hormones with the occurrence of thyroid nodules by gender. The unrestricted cubic splines show that there is no different relation tendency between males and females.

### 2.6. Statistical Analysis

Quantitative variables are expressed as means ± Standard Deviation (SD), and the qualitative variables are presented as percentages. Statistical comparisons were performed by the mean of independent sample *t*-tests for data with a normal distribution, except for TSH, TPOAb, and TGAB, in which non-parametric statistics were used with the Wilcoxon test. The chi-square test was used for categorical variables. After adjustment for potential confounders (including age, sex, BMI, place of residence, smoking, alcohol drinking, salt appetite, types of salt, dietary patterns, the associations of thyroid hormones (T3, FT3, T4, FT4), and thyroid autoantibodies (TPOAb and TGAb)), the risk of thyroid nodules was respectively evaluated among all adults and two genders, using multiple logistic regression models. Unrestricted cubic splines were utilized to evaluate the relationships between thyroid hormones and thyroid nodules. The curves of relationships were performed with STATA 11.0 (STATA Corp, College Station, TX, USA). Statistical significance was considered if the *p* value was <0.05. All other statistical analyses were performed with SAS 9.1 (SAS Institute, Inc., Cary, NC, USA).

## 3. Results

### 3.1. The Distributions of Sociodemographic Characteristics and Thyroid Hormones between Thyroid Nodules and Non-Nodules Groups

The characteristics of the study population are depicted in Table 1. A total of 1271 subjects (529 males, 742 females) were included in the final analyses; out of 1271, 402 subjects suffered from thyroid nodules. Patients with thyroid nodules had older age, lower educational level, were more likely to be urban residents, were more likely to be married, smoked less and consumed more alcohol than those without nodules. Meanwhile, subjects with thyroid nodules more likely enjoyed unbalanced diet patterns, salty or light appetite, and non-iodized salt, compared with those without thyroid nodules. Additionally, compared with subjects without non-nodules, the subjects with thyroid nodules had higher levels of T3, FT3, T4, FT4 and TPOAb, but lower levels of TSH, and TGAb (Table 2).

### 3.2. The Associations of Thyroid Hormones and Antoantibodies with Thyroid Nodules

The associations of thyroid hormones and thyroid autoantibodies with thyroid nodules were estimated using multivariable linear and logistic regression analysis (Table 3 and Table 4). As shown in Table 3, the risk of thyroid nodes increased with the level of TGAb in the multivariable linear mode. Consistently, a high level of TGAb (TGAb ≥ 400 KU/L) was associated with an increased risk of thyroid nodules among pooled samples (OR = 2.86, 95% CI: 1.49–5.51). A similar association was observed in females (OR = 3.13, 95% CI: 1.53–6.40), but no significant association was observed in males. Further, subjects with a high level of TPOAb (TPOAb ≥ 90 KU/L) were associated with an increased risk of thyroid nodules among pooled samples (OR = 1.51, 95% CI: 0.99–2.30) and females (OR = 1.63, 95% CI: 0.99–2.68, *p* = 0.0538), respectively. A significant relationship was not found between thyroid hormones levels (including T3, FT3, T4, FT4 and TSH) and thyroid nodules.

Additionally, we explored the risk trend using unrestricted cubic splines in STATA. The ORs of thyroid nodes for T3, FT3, T4, FT4 showed a parabola trend, but the low interval of ORs is close to 1. The risk of thyroid nodes was kept stable at different levels of TSH. Interestingly, the risk increased obviously when TGAb ≥ 400 KU/L among pooled samples and females, which was similar with the results of the multivariable linear and logistic regressions.

## 4. Discussion

In this report, the associations of thyroid autoantibodies with thyroid nodules were examined among 1271 Chinese coastal adults using different regression models. As a result, we observed that high levels of TGAb or TPOAb were respectively associated with an increased risk of thyroid nodules, especially among female adults.

TPOAb and TGAb are two important thyroid autoantibodies which are commonly found in patients with thyroid diseases [12]. As shown in some previous studies, TPOAb is correlated with the severity of lymphocytic infiltration and could induce antibody-dependent cell-mediated cytotoxicity [13,14]. Boelaert K. et al. [7] reported that TPOAb was dependently associated with thyroid diseases, but little attention has been paid to whether measuring other thyroid autoantibodies, in addition to TSH, could help predict thyroid nodules in human populations. Therefore, we analyzed whether changes in thyroid ultrasound affected the thyroid autoantibodies values. In the present study, we confirmed that TGAb and TPOAb are two important thyroid autoantibodies to assess the risk of thyroid nodules. Further, a high level of TGAb was positively associated with risk of thyroid nodules. Additionally, our results indicated that a high level of TPOAb might be also related to an increased risk of thyroid nodules. Our results were similar to the findings of M. Parham et al. [15] in Iran. They indicated that the different prevalence of thyroid autoantibodies might explain the wide range of the reported prevalence of thyroid nodules. In addition, Eun Sook Kim et al. [16] reported that TGAb was associated with an increased risk of thyroid cancer in thyroid nodules. Similarly, other studies [17,18] also showed an analogous association with malignancy by considering positive thyroid autoantibodies as a whole, including TPOAb and TGAb. In thyroid cancer, the importance of TGAb measurement was stressed because it could interfere with the measurement of thyroglobulin levels [19]. However, little attention has been paid to whether measuring TGAb could be used to assess the risk of thyroid nodules. TGAb occurs in very high concentrations in patients with Hashimoto’s thyroiditis [20]. Thyroglobulin antibody is directed against thyroglobulin. The development of TGAb is a main hallmark of autoimmune thyroid disease (AITD). Meanwhile, Hashimoto’s thyroiditis (the most common type of autoimmune thyroiditis) is the leading cause of a multinodular glands in the United States [21]. Thus, it is possible that the TGAb increased the risk of thyroid nodules by increasing the risk of AITD. Similarly, in our results, a high level of TPOAb might also be related to the risk of thyroid nodules, because TPOAb is a specific serum marker of AITD [22]. Hence, combined with our results, we suppose that TPOAb or TGAb may play an important role in the relationship between Hashimoto’s thyroiditis and thyroid nodules.

Analogously, as shown in other studies, thyroid nodules are more common in women than in men [23]. Female gender was associated with the presence of both single and multiple nodules. Consistently, in the present study, the significant associations of TPOAb and TGAb with thyroid nodules were only observed in females, but not males. Some researchers believe that women may have a higher risk of developing autoimmune diseases of because their more sophisticated immune systems compared to men’s. Women may have a stronger inflammatory response than men when their immune systems are triggered, and inflammation plays a key role in many autoimmune diseases. While this often results in superior immunity among women, it may also increase the risk of developing autoimmune disorders [24]. Another explanation is that estrogen and progesterone can exacerbate thyroid inflammation [25], which may increase the risk of autoimmune thyroid disease, and thyroid inflammation is also associated with the risk of thyroid nodules. Hormone difference may play an important role in the different susceptibility to thyroid nodule among the two genders.

Previous studies indicated an association between elevated TSH levels and the risk of differentiated thyroid carcinoma in patients with nodular thyroid disease [26,27]. However, similar to the results reported by Carles Zafón et al. [27], we found no relationship between nodules and TSH levels. Few studies have focused on the association between benign thyroid nodules and TSH levels, and this specific relationship may need more deep research. Interestingly, we observed that the prevalence of thyroid nodules was significantly higher in urban settings compared with that of the rural area in the present manuscript. This is consistent with the findings of Benvenga S. et al. [28] and Arena S. et al. [29], and this might be related to different exposure to pollutants.

### Limitation

There are some limitations to this study. The number and mass size of thyroid nodules were not recorded in the investigation. The cross-sectional study might result in possible causal relationships should be interpreted with care. The lack of an international calibration standard for thyroid hormones limits the comparability of different methods. In addition, the association of thyroid hormones with thyroid nodules needs to be confirmed in follow-up studies. Further, for the excluding criteria, the prevalence of thyroid nodules in patients with diagnosed autoimmune thyroiditis cannot be analyzed.

## 5. Conclusions

This is the first report focused on the relationship between thyroid hormones, autoantibodies, and the risk of thyroid nodules in the Chinese population. High levels of thyroid autoantibodies may be positively associated with a high prevalence of thyroid nodules among Chinese adults, especially among female adults.

## Figures and Tables

**Table 1:** The distributions of sociodemographic characteristics among patients with and without thyroid nodules.

Variables	Nodules (*n* = 402)	Non-Nodules (*n* = 869)	*p* Value
**Age**	50.53 ± 14.44	48.09 ± 14.46	0.005
**Gender**			
Male	156 (38.81)	373 (42.92)	0.166
Female	246 (61.19)	496 (57.08)	
**Education**			
Primary school *	27 (12.74)	14 (6.86)	0.007
Junior high school	72 (33.96)	48 (23.53)	
Senior high school **	77 (36.32)	99 (48.53)	
Junior college and above	36 (16.98)	43 (21.08)	
**Place of residence**			
Urban area	250 (62.19)	251 (28.88)	<0.001
Rural area	152 (37.81)	618 (71.12)	
**Marriage**			
Unmarried	16 (6.40)	48 (19.12)	<0.001
Married	207 (82.80)	181 (72.11)	
Other	27 (10.80)	22 (8.77)	
**Cigarette smoking**			
Never	346 (86.07)	740 (85.16)	0.005
Ever	6 (1.49)	1 (0.11)	
Current	50 (12.44)	128 (14.73)	
**Alcohol drinking**			
No	376 (93.53)	836 (96.20)	0.0354
Yes	26 (6.47)	33 (3.80)	
**Diet patterns**			
Balanced	368(91.54)	842 (96.89)	<0.001
Vegetarian	26(6.47)	20 (2.30)	
Meat	8(1.99)	7 (0.81)	
**Salt appetite**			
Moderate	321 (79.85)	779 (89.64)	<0.0001
Salty	39 (9.70)	35 (4.03)	
Light	42 (10.45)	55 (6.33)	
Types of salt ***			
Type I	388 (96.76)	855 (98.50)	0.041
Type II	13 (3.24)	13 (1.50)	

* primary school and illiteracy; ** senior high school and technical secondary school; *** Type I = iodized salt consistently, Type II = non-iodized salt consistently or both iodized salt and non-iodized salt.

**Table 2:** The characteristics of thyroid hormone among patients with and without thyroid nodules.

Thyroid Hormone	Nodules (*n* = 402)	Non-Nodules (*n* = 869)	*p* Value
T3 (Mean ± SD)	1.63 ± 0.52	1.38 ± 0.52	<0.001
FT3 (Mean ± SD)	4.06 ± 1.13	3.55 ± 1.17	<0.001
T4 (Mean ± SD)	102.03 ± 24.27	92.34 ± 23.50	<0.001
FT4 (Mean ± SD)	16.68 ± 3.60	13.99 ± 3.25	<0.001
TSH (median (quartile range))	1.87 (1.49)	2.01 (1.57)	0.0532 *
TPOAb (median (quartile range))	13.35 (10.71)	12.49 (11.30)	0.0323 *
TGAb (median (quartile range))	21.10 (22.36)	26.70 (16.85)	0.0004 *

* Wilcoxon test was used for the value.

**Table 3:** Multiple linear regression * to estimate the correlation of thyroid nodules with thyroid hormones and autoantibodies among adults, respectively.

Variables	β	Std Error	*p* Value
**T3**			
	**Pooled**		
Node	−0.0154	0.0233	0.5092
	**Male**		
	−0.0263	0.0409	0.5198
	**Female**		
	−0.0031	0.0281	0.9117
**FT3**			
	**Pooled**		
Node	−0.0643	0.0521	0.218
	**Male**		
	−0.1235	0.0926	0.1828
	**Female**		
	−0.0054	0.0613	0.9295
**T4**			
	**Pooled**		
Node	−0.9463	1.1200	0.4305
	**Male**		
	−1.0416	1.7705	0.5566
	**Female**		
	−0.5843	1.6402	0.7218
**FT4**			
	**Pooled**		
Node	−0.0664	0.1488	0.6556
	**Male**		
	−0.2822	0.2105	0.1808
	**Female**		
	0.1274	0.2078	0.5400
**TSH**			
	**Pooled**		
Node	−0.1465	0.1678	0.3835
	**Male**		
	−0.1011	0.1363	0.4581
	**Female**		
	−0.1678	0.2711	0.5363
**TPOAb**			
	**Pooled**		
Node	7.158	15.686	0.6482
	**Male**		
	12.01	21.056	0.5686
	**Female**		
	5.906	22.455	0.7926
**TGAb**			
	**Pooled**		
Node	41.444	9.712	<0.0001
	**Male**		
	7.701	6.673	0.2493
	**Female**		
	63.4636	15.679	<0.0001

* Adjustment for age, sex, place of residence, smoking, alcohol drinking, salt appetite, types of salt, dietary patterns.

**Table 4:** Logistic regression to estimate the correlation between thyroid hormones and thyroid nodules among adults.

Hormones #	Nodules	Non-Nodules	Adjusted OR (95% CI) *	*p* Value
	**Male**			
**T3** (μg/L)				
Low	59 (39.60)	227 (63.06)	1.00	
High	90 (60.40)	133 (36.94)	1.39 (0.74, 2.62)	0.3030
**FT3** (ng/L)				
Low	48 (37.80)	165 (56.51)	1.00	
High	79 (62.20)	127 (43.49)	1.04 (0.92, 1.19)	0.5147
**T4** (μg/L)				
Low	83 (53.21)	262 (70.24)	1.00	
High	73 (46.79)	111 (29.76)	2.26 (0.91, 5.60)	0.0782
**FT4** (ng/L)				
Low	101 (64.74)	302 (80.97)	1.00	
Medium	41 (26.28)	47 (12.60)	0.99 (0.50, 1.93)	0.9589
High	14 (8.97)	24 (6.43)	0.73 (0.31, 1.75)	0.4837
**TSH** (mIU/L)				
Low	141 (90.38)	327 (87.67)		
Medium	9 (5.77)	37 (9.92)	0.60 (0.27, 1.32)	0.2027
High	6 (3.85)	9 (2.41)	1.29 (0.41, 4.07)	0.6587
**TPOAb** (KU/L)				
Low	147 (94.23)	353 (94.64)	1.00	
High	9 (5.77)	20 (5.36)	1.38 (0.60, 3.21)	0.4595
**TGAb** (KU/L)				
Low	153 (98.08)	370 (99.20)	1.00	
High	3 (1.92)	3 (0.80)	1.05 (0.09, 11.94)	0.9703
	**Female**			
**T3** (μg/L)				
Low	66 (29.07)	291 (61.26)	1.00	
High	161 (70.93)	184 (38.74)	1.23 (0.67, 2.25)	0.5005
**FT3** (ng/L)				
Low	53 (26.50)	247 (61.90)	1.00	
High	147 (73.50)	152 (38.10)	0.86 (0.36, 2.06)	0.7403
**T4** (μg/L)				
Low	119 (48.37)	337 (67.94)	1.00	
High	127 (51.63)	159 (32.06)	0.79 (0.52, 1.21)	0.2791
**FT4** (ng/L)				
Low	156 (63.41)	412 (83.06)	1.00	
Medium	67 (27.24)	63 (12.70)	1.05 (0.66, 1.67)	0.8490
High	23 (9.35)	21 (4.23)	1.16 (0.59, 2.28)	0.6675
**TSH** (mIU/L)				
Low	204 (82.93)	396 (79.84)	1.00	
Medium	30 (12.10)	74 (14.92)	0.80 (0.49, 1.300)	0.3609
High	12 (4.88)	26 (5.24)	1.01 (0.47, 2.16)	0.9784
**TPOAb** (KU/L)				
Low	212 (86.18)	436 (87.90)	1.00	
High	34 (13.82)	60 (12.10)	1.63 (0.99, 2.68)	0.0538
**TGAb** (KU/L)				
Low	221 (89.84)	481 (96.98)	1.00	
High	25 (10.16)	15 (3.02)	3.13 (1.53, 6.40)	0.0018
	**Pooled**			
**T3** (μg/L)				
Low	125 (33.24)	518 (62.04)	1.00	
High	251 (66.76)	317 (37.96)	1.28 (0.83, 1.97)	0.2706
**FT3** (ng/L)				
Low	101 (30.89)	412 (59.62)	1.00	
High	226 (69.11)	279 (40.38)	1.00 (0.58, 1.72)	0.9938
**T4** (μg/L)				
Low	202 (50.25)	599 (68.93)	1.00	
High	200 (49.75)	270 (31.07)	0.87 (0.62, 1.20)	0.3867
**FT4** (ng/L)				
Low	257 (63.93)	714 (82.16)	1.00	
Medium	108 (26.87)	110 (12.66)	1.02 (0.70, 1.50)	0.9152
High	37 (9.20)	45 (5.18)	0.88 (0.53, 1.48)	0.6360
**TSH** (mIU/L)				
Low	345 (85.82)	723 (83.20)	1.00	
Medium	39 (9.70)	111 (12.77)	0.74 (0.49, 1.12)	0.1515
High	18 (4.48)	35 (4.03)	1.08 (0.58, 2.01)	0.8148
**TPOAb** (KU/L)				
Low	359 (89.30)	789 (90.79)	1.00	
High	43 (10.70)	80 (9.21)	1.51 (0.99, 2.30)	0.0581
**TGAb** (KU/L)				
Low	374 (93.03)	851 (97.93)	1.00	
High	28 (6.97)	18 (2.07)	2.86 (1.49, 5.51)	0.0017

* Adjustment for age, sex, place of residence, smoking, alcohol drinking, salt appetite, types of salt, dietary patterns. #: The level was divided by the hormone-OR figure of each kind of hormone. T3: low: T3 ≤ 1.3 μg/L, high: T3 > 1.3 μg/L; FT3: low: FT3 ≤ 3.4 ng/L, high: FT3 > 3.4 ng/L; T4: low: T4 ≤ 100 μg/L, high: T4 > 100 μg/L; FT4: low: FT4 < 17 ng/L, medium: 17 < FT4 < 20 ng/L; high: FT4 ≥ 20 ng/L; TSH: low: TSH < 3.5 mIU/L, medium: 3.5 < TSH < 5.5 mIU/L; high: TSH ≥ 5.5 mIU/L; TPOAb: low: TPOAb < 90 KU/L, high: TPOAb ≥ 90 KU/L; TGAb: low: TGAb < 400 KU/L, high: TGAb ≥ 400 KU/L.

## Supplementary Materials

The following are available online at www.mdpi.com/1660-4601/14/7/723/s1, Figure S1: Unrestricted cubic splines with unadjusted odds ratios plotted for each level of F3; Figure S2: Unrestricted cubic splines with unadjusted odds ratios plotted for each level of FT3; Figure S3: Unrestricted cubic splines with unadjusted odds ratios plotted for each level of T4; Figure S4: Unrestricted cubic splines with unadjusted odds ratios plotted for each level of FT4; Figure S5: Unrestricted cubic splines with unadjusted odds ratios plotted for each level of TSH; Figure S6: Unrestricted cubic splines with unadjusted odds ratios plotted for each level of TPOAb; Figure S7: Unrestricted cubic splines with unadjusted odds ratios plotted for each level of TGAb.
