# Supplementary material for: The Relationship of TPOAb and TGAb with Risk of Thyroid Nodules: A Large Epidemiological Study
Source: Int J Environ Res Public Health. 2017 Jul 5;14(7):723. doi: 10.3390/ijerph14070723 (PMC5551161; doi:10.3390/ijerph14070723)
Supplement: Supplementary file 1 [file ijerph-14-00723-s001.pdf]

International Journal of
Environmental Research and Public Health

Supplementary Materials

The Relationship of TPOAb and TGAb with Risk of Thyroid Nodules: A Large Epidemiological Study

Weimin Xu, LiangliangHuo, Zexin Chen, Yangmei Huang, XingyiJin, Jing Deng, Sujuan Zhu, Yunxian Yu

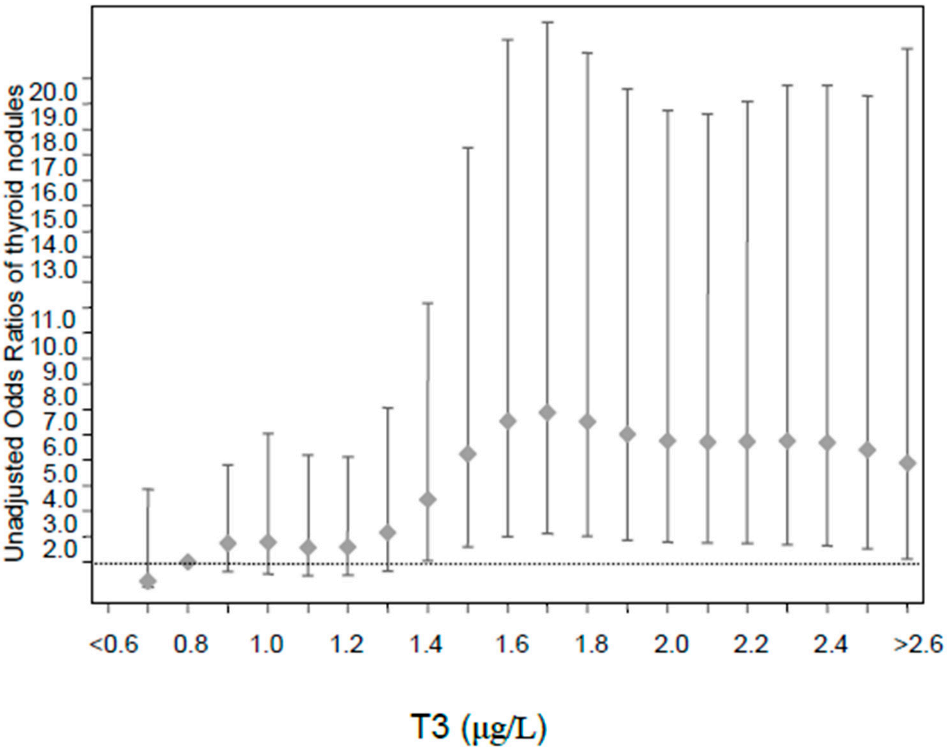

(a)

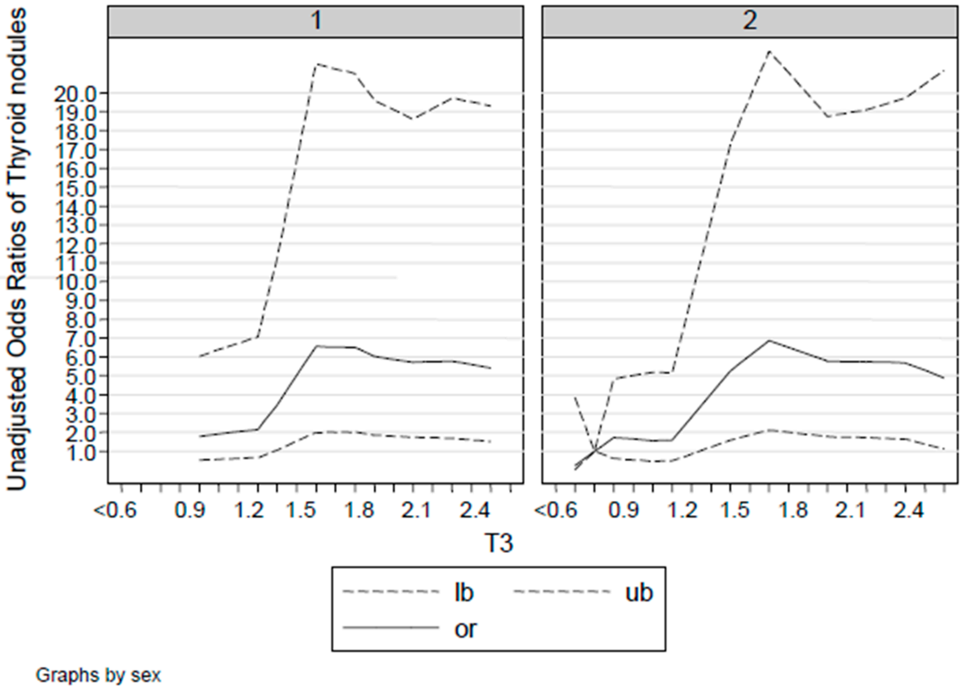

(b)

Figure S1. Unrestricted cubic splines with unadjusted odds ratios plotted for each level of T3. (a) This graph shows unadjusted ORs with 95% CI (capped spikes) for the relation of T3 to the occurrence of thyroid nodules in Chinese adult. T3 was modeled by unrestricted cubic splines with four knots at percentiles 20%, 40%, 60%, and 80% in a logistic regression model. The reference value is level 2. The level of T3 rang from level 1 to level 21. Level 1 represent the concentration under 0.6μg/L, the level 21 represent the concentration over 2.6μg/L. The gap between two levels was 0.1μg/L. (**b**) This graph shows unadjusted ORs with 95% CI (capped spikes) for the relation of T3 to the occurrence of thyroid nodules in males and females of Chinese adult. The small figure1 shows the relation in males; the small figure2 shows the relation in females. T3 was modeled by unrestricted cubic splines with four knots at percentiles 20%, 40%, 60%, and 80% in a logistic regression model. The reference value is level 2. The level of T3 rang from level 1 to level 21. Level 1 represent the concentration under 0.6μg/L, the level 21 represent the concentration over 2.6μg/L. The gap between two levels was 0.1μg/L.

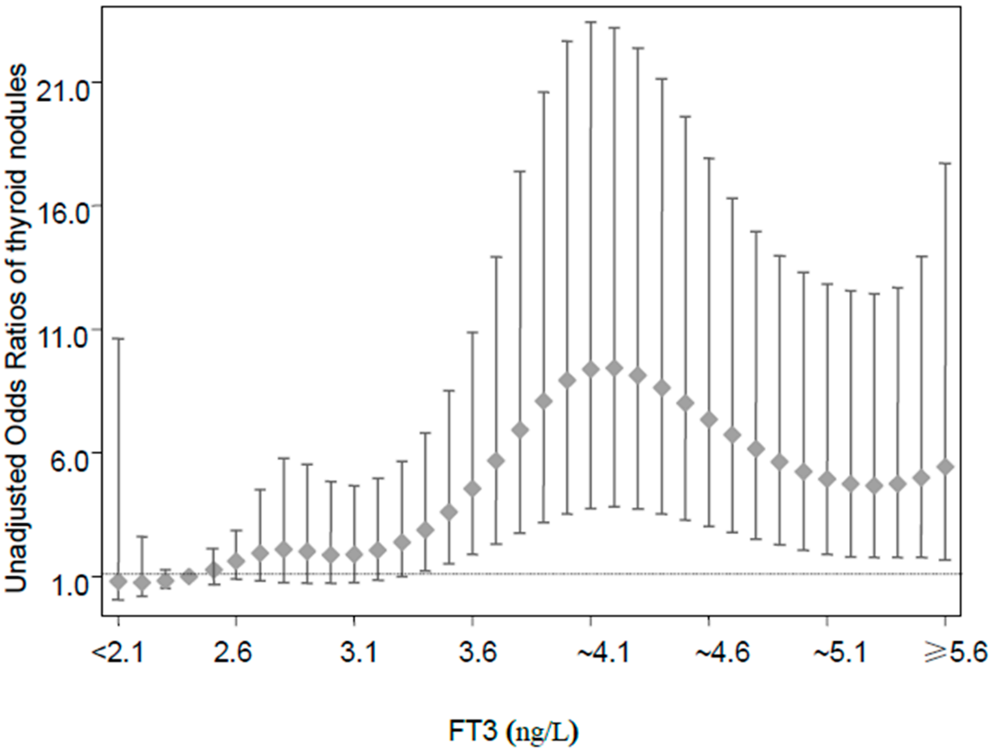

(a)

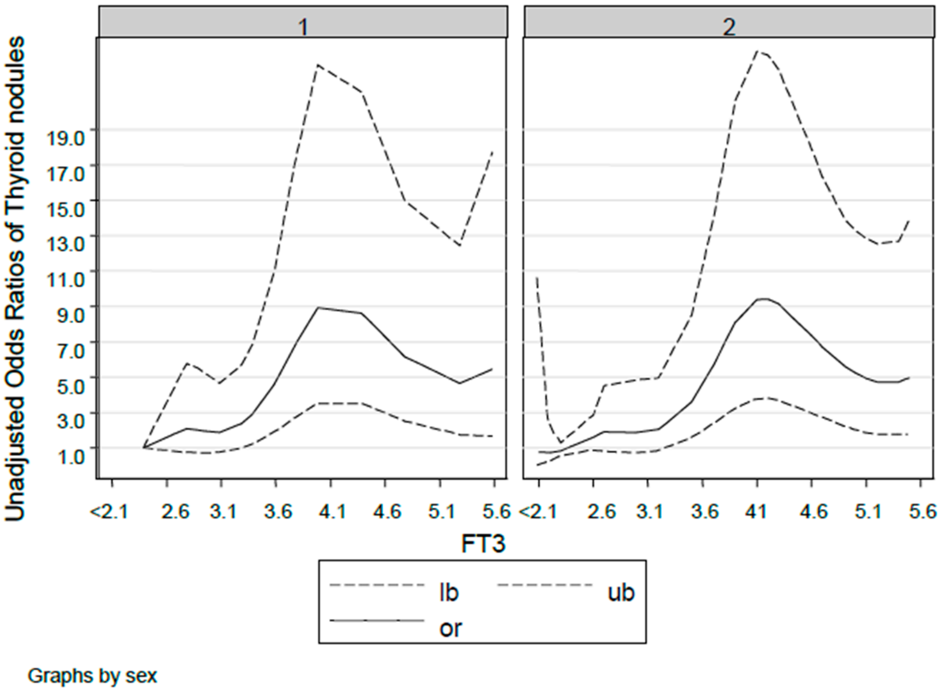

(b)

Figure S2. Unrestricted cubic splines with unadjusted odds ratios plotted for each level of FT3. (**a**) This graph shows unadjusted ORs with 95% CI (capped spikes) for the relation of FT3 to the occurrence of thyroid nodules in Chinese adult. FT3 was modeled by unrestricted cubic splines with four knots at percentiles 20%, 40%, 60%, and 80% in a logistic regression model. The reference value is level 3. The level of FT3 rang from level 1 to level 36. Level 1 represent the concentration between2.1μg/L and 2.2ng/L, the level 36 represent the concentration over 5.6ng/L. The gap between two levels was 0.1ng/L. (**b**) This graph shows unadjusted ORs with 95% CI (capped spikes) for the relation of FT3 to the occurrence of thyroid nodules in males and females of Chinese adult. The small figure1 shows the relation in males; the small figure2 shows the relation in females. FT3 was modeled by unrestricted cubic splines with four knots at percentiles 20%, 40%, 60%, and 80% in a logistic

regression model. The reference value is level 3. The level of FT3 rang from level 1 to level 36. Level 1 represent the concentration between2.1μg/L and 2.2ng/L, the level 36 represent the concentration over 6.0ng/L. The gap between two levels was 0.1ng/L.

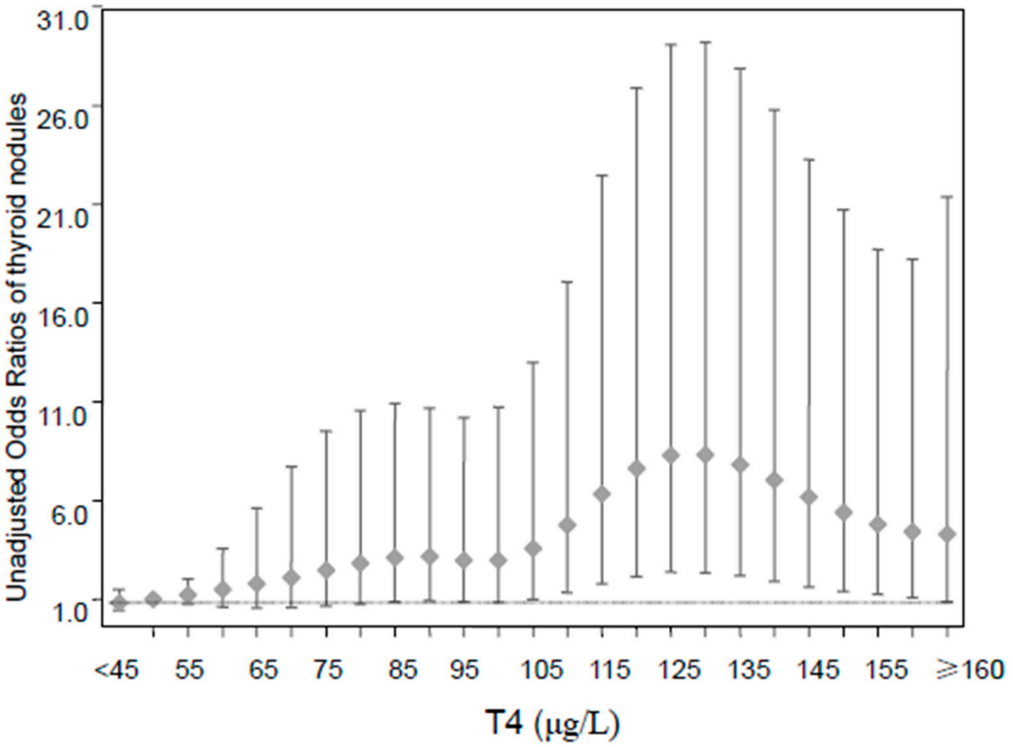

(a)

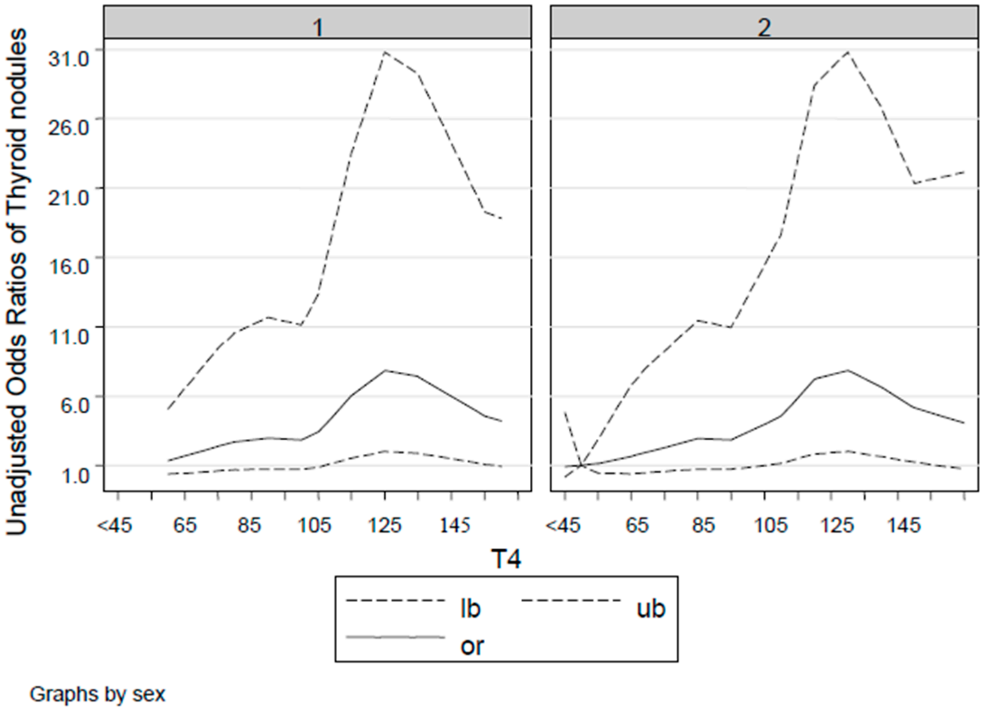

(b)

Figure S3. Unrestricted cubic splines with unadjusted odds ratios plotted for each level of T4. (**a**) This graph shows unadjusted ORs with 95% CI (capped spikes) for the relation of T4 to the occurrence of thyroid nodules in Chinese adult. T4 was modeled by unrestricted cubic splines with four knots at percentiles 20%, 40%, 60%, and 80% in a logistic regression model. The reference value is level 2. The level of T4 rang from level 1 to level 25. Level 1 represent the concentration under 45μg/L, the level

25 represent the concentration over 160μg/L. The gap between two levels was 5μg/L. (**b**) This graph shows unadjusted ORs with 95% CI (capped spikes) for the relation of T4 to the occurrence of thyroid nodules in males and females of Chinese adult. The small figure1 shows the relation in males; the small figure2 shows the relation in females. T4 was modeled by unrestricted cubic splines with four knots at percentiles 20%, 40%, 60%, and 80% in a logistic regression model. The reference value is level 2. The level of T4 rang from level 1 to level 25. Level 1 represent the concentration under 45μg/L, the level 25 represent the concentration over 160μg/L. The gap between two levels was 5μg/L.

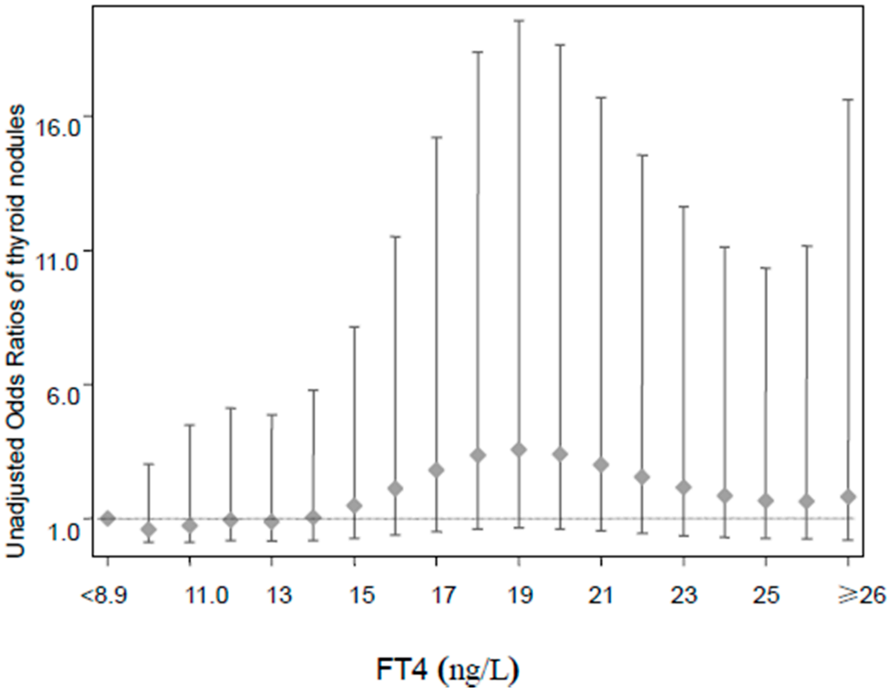

(a)

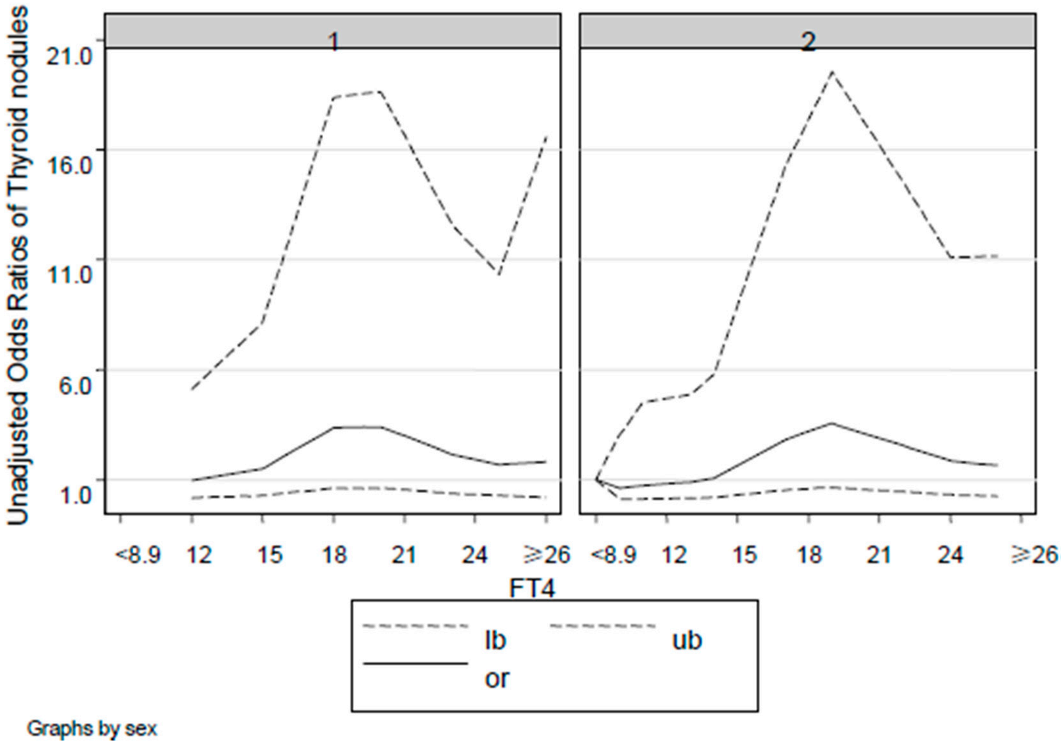

(b)

Figure S4. Unrestricted cubic splines with unadjusted odds ratios plotted for each level of FT4. (**a**) This graph shows unadjusted ORs with 95% CI (capped spikes) for the relation of FT4 to the

occurrence of thyroid nodules in Chinese adult. FT4 was modeled by unrestricted cubic splines with four knots at percentiles 20%, 40%, 60%, and 80% in a logistic regression model. The reference value is level 1. The level of FT4 rang from level 1 to level 19. Level 1 represent the concentration under 8.9ng/L, the level 19 represent the concentration over 26ng/L. The gap between two levels was 1ng/L. (**b**) This graph shows unadjusted ORs with 95% CI (capped spikes) for the relation of FT4 to the occurrence of thyroid nodules in males and females of Chinese adult. The small figure1 shows the relation in males; the small figure2 shows the relation in females. FT4 was modeled by unrestricted cubic splines with four knots at percentiles 20%, 40%, 60%, and 80% in a logistic regression model. The reference value is level 1. The level of FT4 rang from level 1 to level 19. Level 1 represent the concentration under 8.9ng/L, the level 19 represent the concentration over 26ng/L. The gap between two levels was 1ng/L.

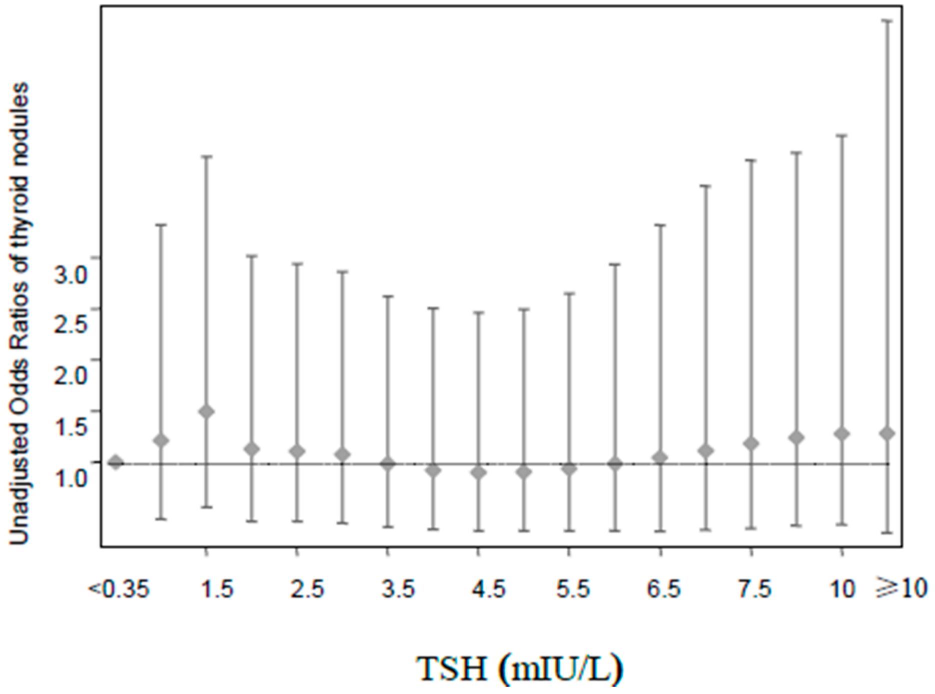

(a)

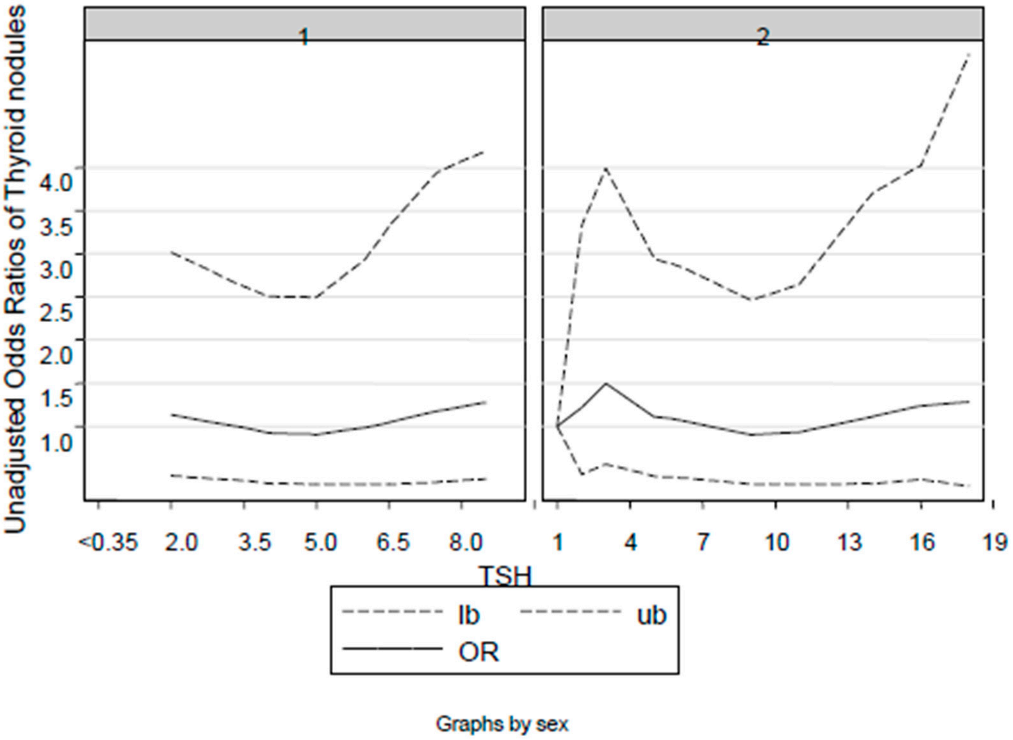

(b)

Figure S5. Unrestricted cubic splines with unadjusted odds ratios plotted for each level of TSH. (**a**) This graph shows unadjusted ORs with 95% CI (capped spikes) for the relation of TSH to the occurrence of thyroid nodules in Chinese adult. TSH was modeled by unrestricted cubic splines with four knots at percentiles 20%, 40%, 60%, and 80% in a logistic regression model. The reference value is level 1. The level of TSH rang from level 1 to level 18. Level 1 represent the concentration under 0.35mIU//L, the level 18 represent the concentration over 10mIU/L. The gap between two levels was 0.5mIU/ L. (**b**) This graph shows unadjusted ORs with 95% CI (capped spikes) for the relation of TSH to the occurrence of thyroid nodules in males and females of Chinese adult. The small figure1 shows the relation in males; the small figure2 shows the relation in females. TSH was modeled by unrestricted cubic splines with four knots at percentiles 20%, 40%, 60%, and 80% in a logistic regression model. The reference value is level 1. The level of TSH rang from level 1 to level 18. Level 1 represent the concentration under 0.35mIU//L, the level 18 represent the concentration over 10mIU/L. The gap between two levels was 0.5mIU/ L.

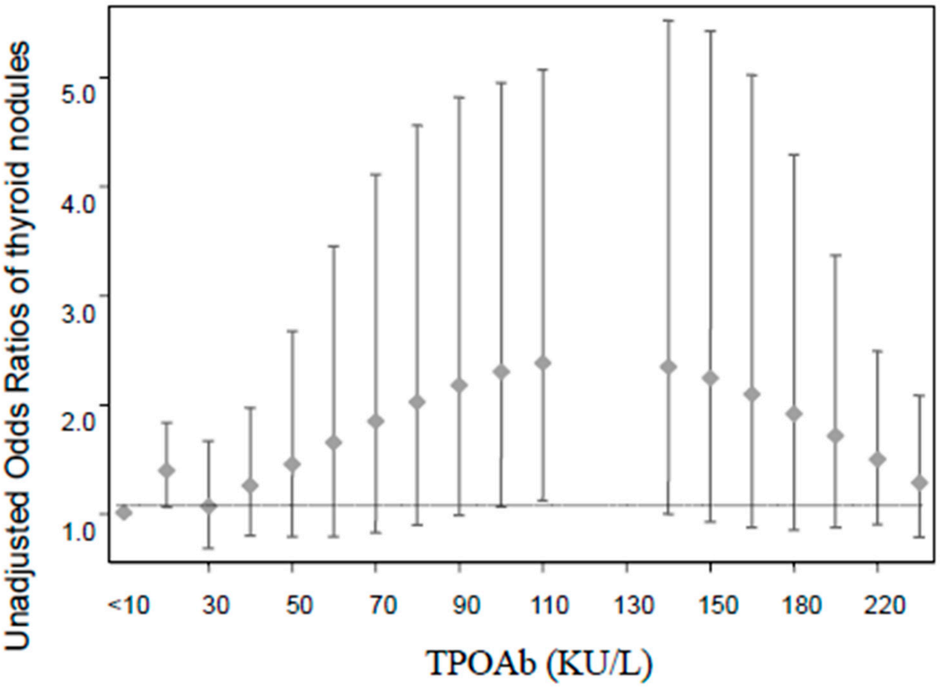

(a)

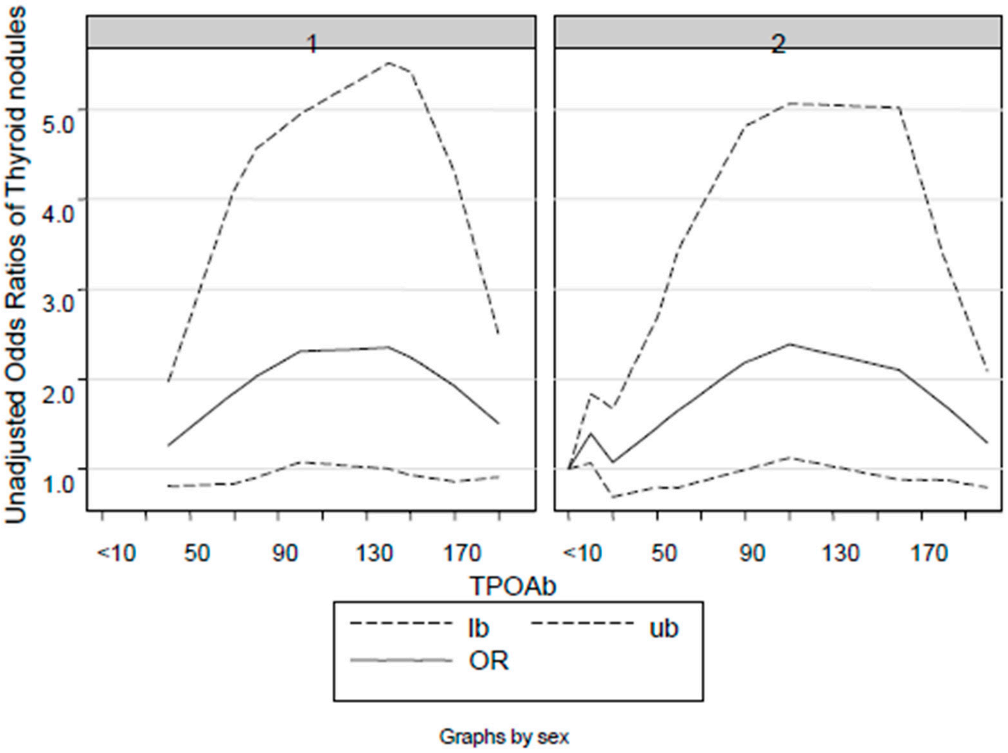

(b)

Figure S6. Unrestricted cubic splines with unadjusted odds ratios plotted for each level of TPOAb. (**a**) This graph shows unadjusted ORs with 95% CI (capped spikes) for the relation of TPOAb to the occurrence of thyroid nodules in Chinese adult. TPOAb was modeled by unrestricted cubic splines with four knots at percentiles 20%, 40%, 60%, and 80% in a logistic regression model. The reference value is level 1. The level of TPOAb rang from level 1 to level 20. Level 1 represent the concentration under 10KU//L, the level 20 represent the concentration over 220KU/L. The gap between two levels was 10KU/ L. (**b**) This graph shows unadjusted ORs with 95% CI (capped spikes) for the relation of TPOAb to the occurrence of thyroid nodules in males and females of Chinese adult. The small figure1 shows the relation in males; the small figure2 shows the relation in females. TPOAb was modeled by unrestricted cubic splines with four knots at percentiles 20%, 40%, 60%, and 80% in a logistic regression model. The reference value is level 1. The level of TPOAb rang from level 1 to level 20. Level 1 represent the concentration under 10KU//L, the level 20 represent the concentration over 220KU/L. The gap between two levels was 10KU/ L.

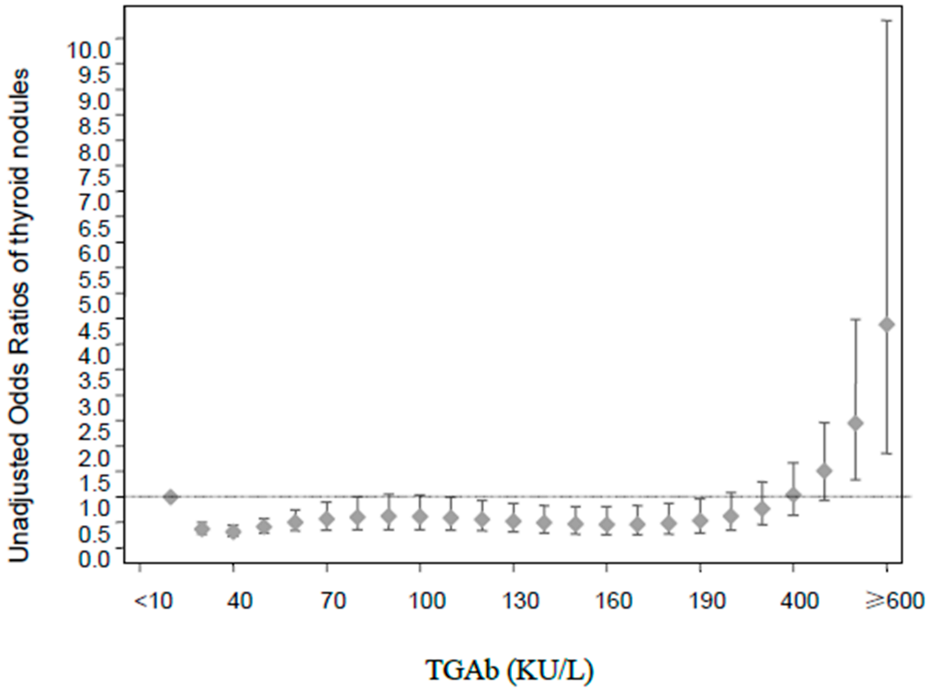

(a)

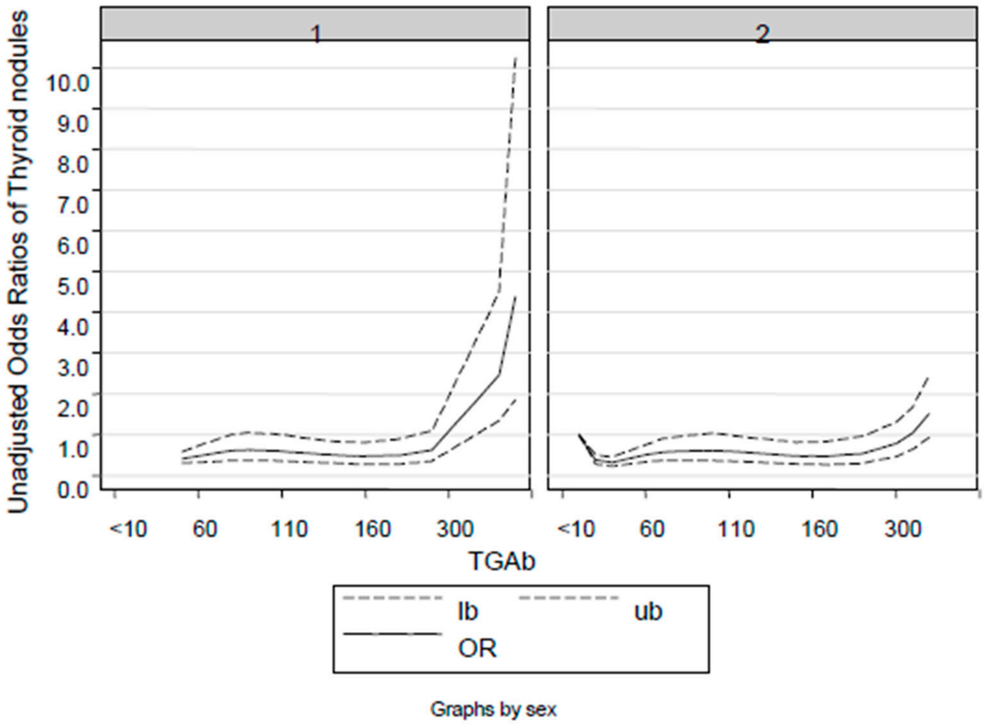

(b)

Figure S7. Unrestricted cubic splines with unadjusted odds ratios plotted for each level of TGAb. (**a**) This graph shows unadjusted ORs with 95% CI (capped spikes) for the relation of TGAb to the occurrence of thyroid nodules in Chinese adult. TPAb was modeled by unrestricted cubic splines with four knots at percentiles 20%, 40%, 60%, and 80% in a logistic regression model. The reference value is level 1. The level of TGAb rang from level 1 to level 25. Level 1 represent the concentration under 10KU//L, the level 20 represent the concentration over 600KU/L. The gap between two levels was 10KU/ L before level 21. The gap between two levels was 100KU/ L from 200KU/L. (**b**) This graph shows unadjusted ORs with 95% CI (capped spikes) for the relation of TGAb to the occurrence of thyroid nodules in males and females of Chinese adult. The small figure1 shows the relation in males; the small figure2 shows the relation in females. TPAb was modeled by unrestricted cubic splines with four knots at percentiles 20%, 40%, 60%, and 80% in a logistic regression model. The reference value

is level 1. The level of TGAb rang from level 1 to level 25. Level 1 represent the concentration under 10KU//L, the level 20 represent the concentration over 600KU/L. The gap between two levels was 10KU/ L before level 21. The gap between two levels was 100KU/ L from 200KU/L.
